# Draft Genome Sequences of Novel *Pseudomonas*, *Flavobacterium*, and *Sediminibacterium* Species Strains from a Freshwater Ecosystem

**DOI:** 10.1128/genomeA.00009-18

**Published:** 2018-02-01

**Authors:** Federica Pinto, Adrian Tett, Federica Armanini, Francesco Asnicar, Adriano Boscaini, Edoardo Pasolli, Moreno Zolfo, Claudio Donati, Nico Salmaso, Nicola Segata

**Affiliations:** a Centre for Integrative Biology, University of Trento, Trento, Italy; b Edmund Mach Foundation, San Michele all’Adige, Italy

## Abstract

Freshwater ecosystems represent 0.01% of the water on Earth, but they support 6% of global biodiversity that is still mostly uncharacterized. Here, we describe the genome sequences of three strains belonging to novel species in the *Pseudomonas*, *Flavobacterium*, and *Sediminibacterium* genera recovered from a water sample of Lake Garda, Italy.

## GENOME ANNOUNCEMENT

The uncharacterized microbial genetic diversity in natural environments is immense. Although high-throughput sequencing methods (1) and metagenomics (2) are recovering part of this diversity, ecosystems, such as freshwater habitats, which are estimated to support 6% of global biodiversity, remain poorly characterized (3). It is thus important to continue uncovering and analyzing microbial sequences from these environments.

In this project, we recovered and described three new microbial genomes of organisms inhabiting a pre-Alpine freshwater lake (Lake Garda, Italy). We specifically focused on potential symbionts of cyanobacterial organisms, and the genomic DNA was obtained from a nonaxenic culture of *Tychonema bourrellyi* (4). Paired-end libraries (Illumina) were prepared and run on the Illumina HiSeq 2500 platform (100-nucleotide [nt]-long paired-end reads). Raw reads were assembled using metaSPAdes version 3.10.1 (5) with default parameters, which generated 7,029 contigs larger than 1,000 bp with a total size of 74 Mbp. From this metagenomic assembly, three genomes belonging to the *Pseudomonas*, *Flavobacterium*, and *Sediminibacterium* genera were binned using the manually supervised anvi’o protocol based on the abundance and tetranucleotide frequency distributions (6) and quality controlled by CheckM (7) (100%, 99%, and 99% predicted completeness, respectively, with an indication of contamination only for *Pseudomonas* at 0.27% and no strain heterogeneity).

The *Pseudomonas* sp. strain FEMGT703P genome has 4.41 Mb assembled into 14 contigs, with an *N*_50_ of 398,350 bp and a GC content of 59.93%. The assembly of the *Flavobacterium* sp. strain FEMGT703F genome resulted in 2.98 Mb, with an *N*_50_ of 372,530 bp and a GC content of 38.68%. The *Sediminibacterium* sp. strain FEMGT703S genome has 3.22 Mb, with an *N*_50_ of 530,195 bases and a GC content of 35.78%. The genomes were annotated using Prokka version 1.11 (8), which identified 4,064, 2,649, and 2,877 coding sequences for the *Pseudomonas*, *Flavobacterium*, and *Sediminibacterium* genomes, respectively. High-resolution phylogenetic profiling using PhyloPhlAn (9) and sequence similarity analysis using pyani (version 0.2.6; option “-m ANIb”) both confirmed that the three genomes belong to new species when using a sequence identity cutoff of 95%.

The discovery of these new three microbial genomes in a nonaxenic *T. bourrellyi* culture might confirm the establishment of an ecological association between cyanobacteria and heterotrophic microbes (10, 11). However, more investigations are still needed to further characterize the microbial community diversity and interactions in freshwater systems.

### Accession number(s).

The sequences for this genome project have been deposited in GenBank under the accession no. PGCM00000000 (*Pseudomonas* sp. FEMGT703P), PGCN00000000 (*Flavobacterium* sp. FEMGT703F), and PGCO00000000 (*Sediminibacterium* sp. FEMGT703S). The versions described in this paper are the first versions, PGCM01000000, PGCN01000000, and PGCO01000000, respectively.
