# Correction for Pinto et al., “Draft Genome Sequences of Novel Pseudomonas, Flavobacterium, and Sediminibacterium Strains from a Freshwater Ecosystem”

**DOI:** 10.1128/genomeA.00169-18

**Published:** 2018-03-08

**Authors:** Federica Pinto, Adrian Tett, Federica Armanini, Francesco Asnicar, Adriano Boscaini, Edoardo Pasolli, Moreno Zolfo, Claudio Donati, Nico Salmaso, Nicola Segata

**Affiliations:** a Centre for Integrative Biology, University of Trento, Trento, Italy; b Edmund Mach Foundation, San Michele all’Adige, Italy

## AUTHOR CORRECTION

Volume 6, no. 5, e00009-18, 2018, https://doi.org/10.1128/genomeA.00009-18. Page 1: The article title should read as given above.

